# Principles of Operation and Application Extensions of Triboelectric Nanogenerators: Structure and Material Optimization

**DOI:** 10.3390/mi16101127

**Published:** 2025-09-30

**Authors:** Li Tao, Tianyu Chen, Jiale Wu, Teng Zhang, Lei Shao, Haoliang Zhang, Litao Liu, Hongbo Wu, Tao Chen, Jingdong Ji

**Affiliations:** 1 Department of Robot Engineering, School of Mechanical Engineering, Jiangsu Ocean University, Lianyungang 222005, China; 13775564826@163.com (T.C.); 2023210507@jou.edu.cn (J.W.); 19850220514@163.com (T.Z.); 17388439895@163.com (L.S.); a15890744742@163.com (H.Z.); liulitao99@163.com (L.L.); 2 The New Energy Limited Company of Xu Zhou Bo Xu, E1-401, National Security Technology Industrial Park, Xuzhou High-Tech Industrial Development Zone, Xuzhou 221000, China; wuhongbo0718@163.com (H.W.); chentao202507@163.com (T.C.); jijing09142025@163.com (J.J.)

**Keywords:** triboelectric nanogenerator (TENG), tribometric mechanisms, nanotechnology, energy conversion

## Abstract

As research on triboelectric nanogenerators (TENGs) continues to advance, their applications are becoming increasingly diverse and sophisticated. This paper aims to provide future researchers with a concise yet comprehensive understanding of the four fundamental operational principles of TENGs, enabling them to fully appreciate the unique characteristics and application scenarios of each mode. In doing so, researchers can make informed and well-grounded choices in selecting the most suitable operational mode for exploration and innovation, tailored to their specific fields and requirements. Furthermore, this paper aligns closely with the current research frontiers and development trends of TENGs by systematically reviewing the literature and analyzing recent developments in the field from three key perspectives: the expansion of application domains, innovations in structural design, and optimizations in material properties. Through this multidimensional framework, it not only highlights the broad potential and practical prospects of TENGs but also uncovers the latest advancements and future directions in technological breakthroughs and performance enhancement.

## 1. Introduction

With the continuous advancement of human society, the global demand for energy continues to grow. As a critical factor in improving the quality of human life and promoting the sustainable development of modern society, the importance of energy is self-evident [1]. However, the limited reserves of traditional fossil fuels and the ongoing increase in energy consumption have posed significant challenges to environmental sustainability and resource management [2]. Consequently, the development of sustainable and low-carbon energy technologies, particularly innovative breakthroughs in renewable energy, has become an urgent necessity for ensuring the sustainable development of human civilization [3].

In 2012, Academician Wang Zhonglin and his research team first proposed and developed the Triboelectric Nanogenerator technology, based on the coupling mechanism of the triboelectric effect and electrostatic induction [4]. This technology efficiently converts mechanical energy into electrical energy in various forms [5], such as wind energy, ocean energy, vibration energy, and human motion energy [6]. Compared to traditional electromagnetic induction-based power generation technologies, TENG provides several notable advantages: high output voltage [7], light weight [8], compact size [9], excellent structural flexibility, strong shape adaptability, and outstanding compatibility [10], making it an attractive option for researchers.

TENG is an innovative energy harvesting device, with a fundamental principle based on driving current flow through charge separation and potential differences induced by interfacial friction, pressure, or vibration [11], thus achieving efficient conversion of mechanical energy into electrical energy. TENG can directly harvest energy from the mechanical energy present in the surrounding environment, without requiring an external power source, and exhibits stable operational characteristics [12]. Furthermore, the output power of TENG can be enhanced by coupling it with an electromagnetic generator [13], forming a hybrid triboelectric nanogenerator that efficiently captures various available energy sources [14]. Its inherent flexibility and tunability allow it to adapt to various device geometries and application scenarios [15]. Notably, the power generation performance of TENG can be effectively amplified or adjusted through multiple factors, such as laser cutting power [16], semiconductor surface roughness [17], slider shape, and interfacial medium [18]. These findings offer valuable insights for designing high-performance TENGs. By regulating the surface dielectric constant through physical, chemical, or micropatterning methods, or by increasing the effective contact area or surface roughness [19], the output performance of the device can be optimized.

When selecting triboelectric materials, the material’s ability to attract electrons plays a crucial role in enhancing the electrical performance of TENGs and provides an effective strategy for optimizing device output [20]. Moreover, the seamless integration of TENGs into microdevices and wearable technologies [21], as well as its excellent compatibility in small-scale applications, demonstrates its great potential in the field of efficient and autonomous energy harvesting [22].

This article aims to provide future researchers with a comprehensive yet quick understanding of the four operational principles of triboelectric nanogenerators (TENGs), enabling them to thoroughly grasp the characteristics and application scenarios of these modes. This will aid in their scientific selection of the most suitable operational mode for future research exploration and innovation, based on specific fields and needs. Simultaneously, this article is closely aligned with the current research frontiers and development trends of TENGs by systematically reviewing and analyzing the research landscape from three key perspectives: the expansion of application domains, innovations in structural design, and optimizations in material properties. Through this multidimensional approach, it not only highlights the vast potential of TENGs in practical applications but also uncovers the latest advancements and future directions for technological breakthroughs and performance improvements.

## 2. TENG Working Principle and Working Mode

### 2.1. Origin of TENG Theory

In the mid-19th century, building upon Gauss’s law, Faraday’s law of electromagnetic induction, Ampère’s law, and other contemporary experimental findings, James Clerk Maxwell deduced that the conservation of charge could not be fully explained by existing theories [23]. To address this, he introduced the concept of the displacement current into Maxwell’s equations—a groundbreaking step that provided a theoretical basis for the existence of electromagnetic waves [24]. At the turn of the 20th century, British scientists, drawing on Maxwell’s equations, not only introduced the concept of radio waves but also experimentally confirmed the existence of electromagnetic waves [25].

**Figure 1:**
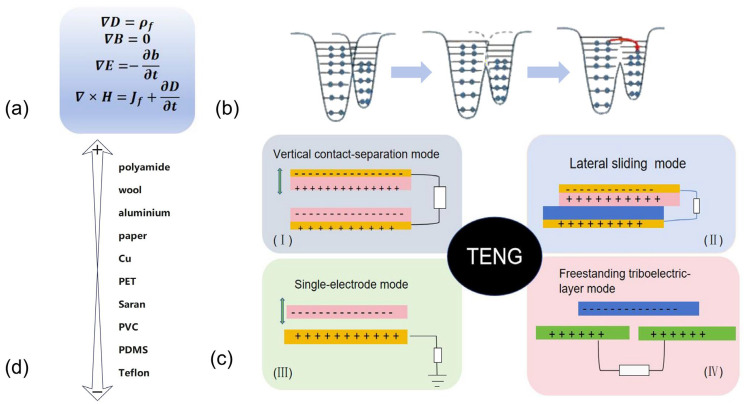
Origin of TENG: (**a**) Maxwell’s Equations; (**b**) Electron Transfer; (**c**) Four Working Modes of TENG; (**c-I**) vertical contact-separation mode striboelectric nanogenerator; (**c-II**) lateral sliding mode triboelectric nanogenerator; (**c-III**) single-electrode mode triboelectric nanogenerator, and (**c-IV**) freestanding triboelectric-layer mode triboelectric nanogenerator; (**d**) Common Materials for Triboelectric Series.

The mathematical formulation of Maxwell’s equations is presented in Figure 1a, where E denotes the electric field, B represents the magnetic field, H signifies the magnetization field, ρ stands for the free charge density, J is the free current density, and D corresponds to the displacement field.

In a serendipitous experiment conducted in 2006, the research group led by Wang Zhonglin discovered that piezoelectric polarization charges, together with the generated time-varying electric field, could drive electron flow through an external circuit. This breakthrough led to the invention of piezoelectric triboelectric nanogenerators (TENGs). After a series of experiments, it was demonstrated that the theoretical foundation of piezoelectric triboelectric nanogenerator is rooted in the Maxwell displacement current, which arises from Maxwell’s four fundamental equations and also underpins the theory of electromagnetic waves.

Further advancements by Wang Zhonglin’s team culminated in the formal introduction of TENGs in 2012. In this work, they identified four distinct working modes, as illustrated in Figure 1c [26]: (c-I) vertical contact-separation mode striboelectric nanogenerator, (c-II) lateral sliding mode triboelectric nanogenerator, (c-III) single-electrode mode triboelectric nanogenerator, and (c-IV) freestanding triboelectric-layer mode triboelectric nanogenerator. Moreover, they developed a triboelectric series to classify electrostatic charge characteristics, as shown in Figure 1d.

### 2.2. Vertical Contact-Separation Mode

**Figure 2:**
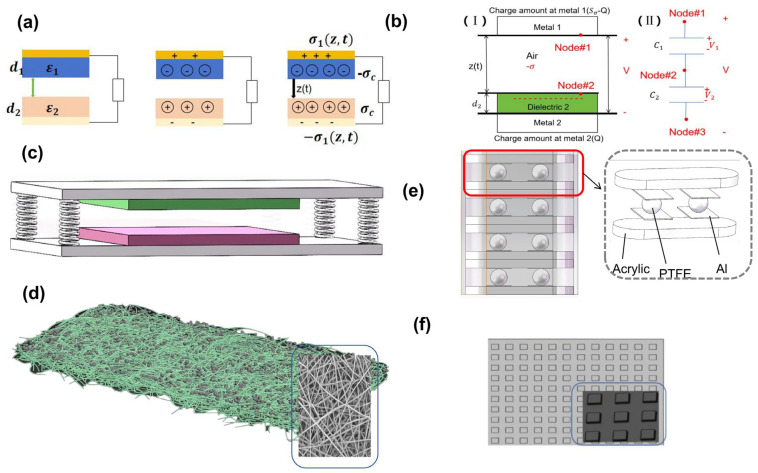
Explanation of Vertical Contact-Separation Mode: (**a**) Schematic diagram of working principle, with friction layers all being dielectric materials as an example; (**b**) Friction layers consisting of both dielectric and conductive materials; (**I**) Friction layers, respectively, being dielectric and conductive materials; (**II**) Attached electrode structure based on (**I**); (**c**) Model of vertical nanogenerator contact spring; (**d**) Friction layer adopting electrospinning structure; (**e**) Multi-layer stacked structure; (**f**) Special treatment applied to the counter-friction layer to give its surface a square-like microstructure.

The vertical contact-separation TENG is the simplest, most basic and most commonly used mode among the four generators. As shown in Figure 2a, we choose dielectric materials with dielectric constants *ε*_1_ and *ε*_2_ and thicknesses $d_{1}$ and $d_{2}$ as the friction layer.

But as the friction begins, and the two dielectric substances contact, at this time, the electrostatic charge begins to transfer to the surfaces of both. The density $\sigma_{c}(t)$ of surface charge increases with the increase in contact times, and finally reaches saturation. The electric fields of the two dielectrics are $E_{Z}=\sigma_{1}(z,t)/\varepsilon_{1}$ and $E_{z}=\sigma_{1}(z,t)/\varepsilon_{2}$, respectively, so the relative voltage difference between the two electrodes at this moment is as follows

(1) $$V=\sigma_{1}(z,t)[d_{1}/\varepsilon_{1}+d_{2}/\varepsilon_{2}]+z[\sigma_{1}(z,t)-\sigma_{c}]/\varepsilon_{0}$$

Under short-circuit condition, V = 0,

(2) $$\sigma_{1}(z,t)=\frac{z\sigma_{c}}{d_{1}\varepsilon_{0}/\varepsilon_{1}+d_{2}\varepsilon_{0}/\varepsilon_{2}+z}$$

The displacement current density in the material obtained from Equation (2) is

(3) $$J_{D}=\frac{𝜕D_{Z}}{𝜕t}=\frac{𝜕\sigma_{1}(z,t)}{𝜕t}=\sigma_{c}\frac{dz}{dt}\frac{d_{1}\varepsilon_{0}/\varepsilon_{1}+d_{2}\varepsilon_{0}/\varepsilon_{2}}{{\left[d_{1}\varepsilon_{0}/\varepsilon_{1}+d_{2}\varepsilon_{0}/\varepsilon_{2}+z\right]}^{2}}+\frac{d\sigma_{c}}{dt}\frac{z}{d_{1}\varepsilon_{0}/\varepsilon_{1}+d_{2}\varepsilon_{0}/\varepsilon_{2}+z}$$

According to ohm’s law, the equation of its output current is

(4) $$RA\frac{d\sigma_{1}(z,t)}{dt}=z\sigma_{c}/\varepsilon_{0}-\sigma_{1}(z,t)[d_{1}/\varepsilon_{1}+d_{2}/\varepsilon_{2}+z/\varepsilon_{0}]$$

During experimental investigations, it is impractical for both friction layers in the contact-out mode to consist solely of dielectric materials. Alternative configurations, as illustrated in Figure 2b, include (I) friction layers composed of a dielectric material and a conductive material, respectively, and (II) an electrode structure integrated based on this configuration. With an understanding of the basic working principle, we now examine the working mode, as shown in Figure 2c, which represents a straightforward vertical contact-separation TENG.

Existing studies on vertical contact-separation TENGs often focus on the treatment of friction layer materials, which can be approached in two primary ways: chemical modification of the materials and the use of electrospun structures, as depicted in Figure 2d. A notable example of the former involves improving power generation efficiency by chemically processing two materials to create a novel polymer. In Ref. [27], silk nylon 66 nanofibers were employed to enhance positive charge generation, while polyester was coated with PVDF to improve negative charge friction. Compared to the silk/PET combination, this approach resulted in a 17-fold increase in output voltage density, with a power output of 280 mW/m^2^ at a resistance of 4 mΩ.

Additionally, as shown in Figure 2f, the friction layer can undergo both physical and chemical modifications, as well as micromachining techniques, to increase friction and enhance power generation efficiency [28].

Another physical fabrication approach, illustrated in Figure 2e, utilizes a stacked structure. This technique, as the name suggests, designs a vertical structure assembled through mechanical fixation methods, such as bundling, to increase output power. For example, Xi Liang et al. [29] developed a hexagonal TENG network composed of spherical TENG units based on a spring-assisted multilayer structure. This system was integrated with a power management module (PMM) for harvesting wave energy. The TENG achieved its highest output at a frequency of 1 Hz and an amplitude of 2.5 V, generating a voltage of 354 V, a current of 270 μA, and a power output of 3.33 W/m^3^.

In summary, the vertical contact-separation mode offers a simple and stable structure. Consequently, TENGs based on this operational mode are often structurally enhanced to create repetitive configurations. The inherent simplicity of this structure provides advantages over sliding contact modes in terms of reduced friction and lower material wear. As a result, many researchers use this configuration as a foundation for investigating the effects of material properties on the electrical output performance of TENGs.

### 2.3. Lateral Sliding Mode

This chapter introduces the second fundamental operational mode of TENGs, namely the lateral sliding type. In contrast to the vertical contact-separation mode, the lateral sliding mode does not require an air gap between the two friction surfaces. This feature eliminates the need for an air gap in the design, thereby facilitating the subsequent packaging process. Furthermore, it enables operation in both planar and rotary sliding modes, making it suitable for a wide range of triggering methods [29].

**Figure 3:**
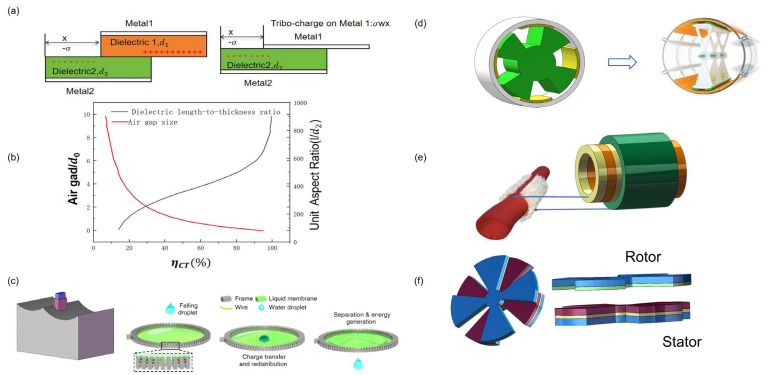
Lateral Sliding Mode: (**a**) Laterally sliding TENG with dielectric–dielectric and conductor-dielectric as friction layers; (**b**) When the friction layer is completely separated, the influence of air gap size and dielectric length-thickness ratio on charge transfer efficiency; (**c**) liquid metal structure [36]; (**d**) rotating cylinder structure; (**e**) encapsulating the tubular structure; (**f**) segmented disk structure.

The underlying mechanism of this mode is based on the coupling effect between sliding friction and in-plane charge separation, driven by an externally applied lateral force. As illustrated in Figure 3a, when an external force is applied, friction between the contact surfaces causes them to slide relative to each other, generating a dense distribution of triboelectric charges. The periodic variation in the contact area leads to lateral separation of the charge centers, which subsequently creates a potential difference. This potential difference drives the flow of electrons through an external load, effectively balancing the triboelectric charges [30].

In the present study, it was observed that the air-gap size and the length-to-thickness ratio of the dielectric exert opposing effects on charge transfer, as shown in Figure 3b. The sliding friction electric sensor is capable of evaluating both the instantaneous and average speeds of each interaction within the contact area, thus enabling real-time monitoring of the tribological behavior during sliding [31]. To optimize output performance and broaden the applicability of lateral sliding TENGs, researchers have proposed several novel structural designs, including liquid-metal structures [32], rotating-disk structures [33], rotating-column structures [34], and encapsulated-tube structures [35]. These innovative configurations significantly enhance both the efficiency and versatility of lateral sliding TENGs.

As illustrated in Figure 3c, TENGs with a liquid metal structure represent an innovative energy-harvesting device that integrates the unique properties of liquid metal with the operational principles of TENGs. This design leverages the high conductivity, exceptional shape adaptability, and fluidity of liquid metal, alongside the triboelectrification effect, to effectively convert mechanical energy into electrical energy. For example, Jinhui Nie et al. [36] demonstrated the ability to harvest mechanical energy using a freely suspended liquid film, without altering the motion of impacting objects such as raindrops, irrigation currents, microfluids, and small particles. Upon the impact of a 40 μL droplet on a pre-charged film, the device generated a peak power of 137.4 nW. Furthermore, In-Yong Suh et al. [37] optimized the performance and application of solid liquid contact electrification triboelectric nanogenerator (SLCE-TENG). By adjusting variables such as the solid surface structure (e.g., nanowire length and surface coatings) and liquid properties (e.g., temperature, ion concentration, and pH), they significantly enhanced both voltage output and current efficiency. SLCE-TENGs have shown great potential for applications in marine energy harvesting, rainwater energy collection, and self-powered sensors. Future development directions for SLCE-TENGs include further enhancement of current output and multifunctional integration to expand their practical applications.

As depicted in Figure 3d, the rotary TENG with a cylindrical structure is employed to harvest rotational mechanical energy, functioning similarly to an triboelectric nanogenerator. The operation of the rotary TENG is based on the coupling of contact-friction electrification and electrostatic induction. The rotating cylinder design exploits the relative sliding motion of a grid surface. Yida Xin et al. [38]. proposed an intelligent triboelectric cylindrical roller bearing (TCRB), in which the cylindrical roller is made of polyether ether ketone (PEEK), and its outer ring consists of grid electrodes coated with a nylon film. The TCRB demonstrated long-term stable operation, with experimental results showing maximum output at 600 rpm, yielding an open-circuit voltage of 26.56 V and a short-circuit current of 2.45 μA. This output was sufficient to drive a small sensor, and the system’s output can be processed for speed monitoring with an error margin of less than 2%.

Figure 3e illustrates a tubular packaging structure that generates energy through reciprocating motion, induced either by direct force or inertia. This TENG type utilizes electrode pairs with finely structured grid electrodes on the sliding surface of the cylinder to generate alternating current. The mechanism involves two coaxial cylinders sliding against each other. Chuyu Tang et al. [39] replaced the middle tubular structure with blood vessels and, through a bionic sheath design, coupled a flexible piezoelectric sensor with a soft, growing artery. This design enabled real-time, high-precision, long-term hemodynamic sensing. Experimental results confirmed the system’s reliability and safety for cardiovascular condition monitoring, providing real-time assessment of cardiovascular health and improving postoperative rehabilitation evaluation for patients with cardiovascular diseases such as aneurysms or atherosclerosis. The “growing” and unconstrained sheath design also holds promise for the development of other bioelectronic devices, potentially improving therapeutic outcomes and patients’ quality of life. Additionally, Changzheng Li et al. [40] proposed a gas-driven triboelectric nanogenerator (GD-TENG) that utilizes liquid column movement for mechanical energy collection and displacement monitoring. In this design, a single liquid column driven by gas within a PTFE tube generates efficient sliding friction at the solid–liquid interface, producing an open-circuit voltage. The performance of the GD-TENG was optimized by systematically studying parameters such as liquid column length, PTFE tube wall thickness, and the speed of liquid column movement. The GD-TENG successfully harvested mechanical energy to power microelectronic devices and a displacement monitoring system for moving objects, offering a new research pathway for self-powered displacement monitoring.

Figure 3f illustrates a rotating TENG with a segmented disk structure, designed to collect energy from rotational motion, particularly in wave energy harvesting applications [40]. The advantage of this design lies in its ability to reduce output voltage while enhancing output current, ensuring that the total output power is not compromised. This design contributes to more efficient energy collection in alignment with green development goals. Yuchen Hu et al. [41] improved the traditional rotating blade design by enabling more intimate interaction between the wave and the device. The improved blade rolls on water like a wheel, continuously stirring the internal TENG. Furthermore, the addition of a superelastic mesh structure allows the blade to expand and contract like a spring, storing wave energy and intensifying the rolling motion of the device. Various synergistic driving modes can thus be realized under combined wave and wind excitation.The comparison of the advantages and disadvantages of four sliding friction based working modes is shown in Table 1.

In summary, compared to the vertical contact mode, the sliding mode offers a broader range of structural design possibilities, enabling a wider array of application scenarios. This expanded design flexibility significantly enhances the potential applications of TENGs. However, the increased sensitivity to sliding contact leads to more frequent contact events, resulting in greater material wear. This trade-off must be carefully considered in the development of more durable and efficient TENG devices.

**Table 1:** Four working modes based on lateral sliding mode.

Lateral Sliding Mode		Practical Application	Pros.	Cons.	Refs.
Rotating Disk Structure	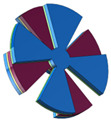	wearable devices, Industrial Monitoring, Environmental energy harvesting	Efficient mechanical energy harvesting through continuous friction and charge separation, compact design, easy integration, and adjustable size and material to accommodate different rotational speeds and energy needs.	Long-term rotation may reduce efficiency due to friction material wear, high requirements for speed and stability, low speed or irregular motion efficiency decreases, and high-speed operation may produce noise and vibration.	[41]
Rotate the cylindrical structure	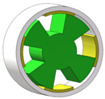	Wave Energy Harvesting, Wind power, Self-powered sensors	The large friction area is used to improve the efficiency of charge generation, adapt to complex dynamic environments such as ocean or wind power, and increase the output power through modular series connection.	The manufacturing process is complex and costly, and long-term operation may increase friction loss due to material fatigue or surface roughness, and it requires more space than the disk structure, which limits the miniaturization application	[42]
Tubular package construction	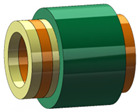	Medical implant devices, Pipeline monitoring, Portable devices,	Encapsulated to protect internal materials, suitable for liquid or wet environments, high durability and long life, and can be designed as flexible or rigid construction to suit a variety of applications.	The limited internal friction area and low output power require a precision packaging process that increases manufacturing costs and requires additional design optimization in the integration of small devices	[43]
Liquid metal construction	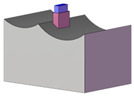	Flexible Electronics Biomedicine Extreme Environments	Adapting to complex shapes and dynamic deformations with high flexibility, excellent conductivity improves charge transfer efficiency and has the potential for self-healing.	Gallium-based alloys are costly, can cause corrosion due to chemical reactions, and require high-precision packaging to prevent leaks, limiting large-scale applications and increasing manufacturing complexity.	[44]

### 2.4. Single-Electrode Mode

**Figure 4:**
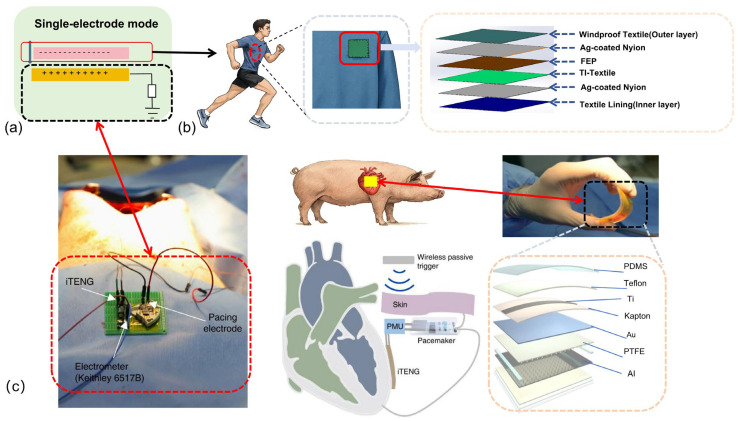
Application of single-electrodes mode: (**a**) Principle of single-electrode mode; (**b**) Application in wearable devices; (**c**) Application in implantable medical monitoring, adapted from [46].

In the study and application of the first two working modes of TENGs, researchers have identified a unique scenario in which one electrode of the device cannot be directly measured, as illustrated in Figure 4a. This configuration is referred to as the single-electrode mode. A representative case arises in wearable applications, where the human body or clothing functions as one of the electrodes, as shown in Figure 4b. In such cases, direct measurement becomes challenging, thereby necessitating the adoption of single-electrode mode. For example, Haishuang Jiao et al. [45] developed a wearable TENG based on thermal insulation textiles (TI-textiles) for advanced health monitoring and multifunctional human–machine interaction. As depicted on the right side of Figure 4a, the device integrates several functional layers, including a triboelectric layer, an Ag-coated nylon electrode, a windproof outer textile layer, and an inner textile layer. In such complex environments, the single-electrode mode not only overcomes the limitations associated with electrode measurement but also establishes a foundation for multi-level functional research.

Beyond wearable applications, the single-electrode mode has also demonstrated remarkable potential in implantable medical monitoring. For example, in cardiac pacemakers implanted near the heart, direct measurement of the electrical current on the cardiac side is impractical, making single-electrode mode the natural operating mode. Han Ouyang et al. [46]. reported a fully implantable symbiotic pacemaker powered by an implantable TENG (iTENG), as shown in Figure 4b. Their work demonstrated that the system could harvest and store biomechanical energy to achieve autonomous cardiac pacing in large animal models. The device effectively corrected sinus arrhythmia and prevented further disease progression. Notably, the implantable TENG achieved an open-circuit voltage of 65.2 V, while the harvested energy per cardiac cycle reached 0.495 μJ, exceeding the pacing threshold energy of 0.377 μJ. These findings confirm both the feasibility and the clinical promise of single-electrode mode in implantable medical devices, offering strong support for the development of next-generation self-powered medical systems. In addition to these representative applications, single-electrode configurations have also been employed across various other domains, as summarized in Table 2.

**Table 2:** Situation and application of several single-electrode mode TENG.

Materials	VOC (V)	ISC (µA)	Applications	Ref.
PVDF/CNC	2	0.155	Sensor	[47]
PVDF/PZT	0.44	-	Vibration energy recovery	[48]
PVDF/KNN nanostructures	1.9	-	Nanomaterials	[49]
PVDF/KNN NRs	17.5	0.522	Wearable devices	[50]
PVDF/BT	1.1	-	Electrospun nanocomposite	[51]
PVDF/rGO/BT	1.2	0.0025	Wearable devices and sensors	[52]
PVDF/SM-KNN NRs	21	22	Electrospun nanocomposite based piezoelectric materials	[51]
PVDF/Ag-Nylon	0.38	1750	Wearable devices	[53]
PVDF-HFP/CNC/Fe-ZnO	12	2.5	Wearable devices	[54]
PVDF/KNN NRs	3.7	0.000326	Nanomaterials	[55]
PVDF-HFP/Co-ZnO	2.8	-	Nanofibers	[56]
PVDF-TrFE/BT NPs	3.4	0.523	Self powered sensor	[57]
PVDF/KNN-ZS	25	2.11	Nanofibers	[58]
PVDF-TrFE/MWCNTs	18.23	2.14	Wearable devices	[59]
PVDF/KNN/CNT	23.24	9	Carbon nanotubes	[60]

### 2.5. Freestanding Triboelectric Layer Mode

Freestanding-layer triboelectric nanogenerators (F-TENGs) are engineered such that the friction layer remains in non-contact with the electrode, thereby eliminating direct wear between the two components. This design significantly extends the operational lifespan of the device [60]. Due to its freestanding, non-contact configuration, this architecture is versatile and can be integrated into a variety of devices, including those featuring sliding structures, rotating wheels, and other similar mechanisms. A TENG functioning in the freestanding-layer mode typically comprises a freestanding layer and a pair of static electrodes. The operational principles of contact electrification and electrostatic induction are employed, where symmetrical electrodes are strategically placed beneath the dielectric layer.

**Figure 5:**
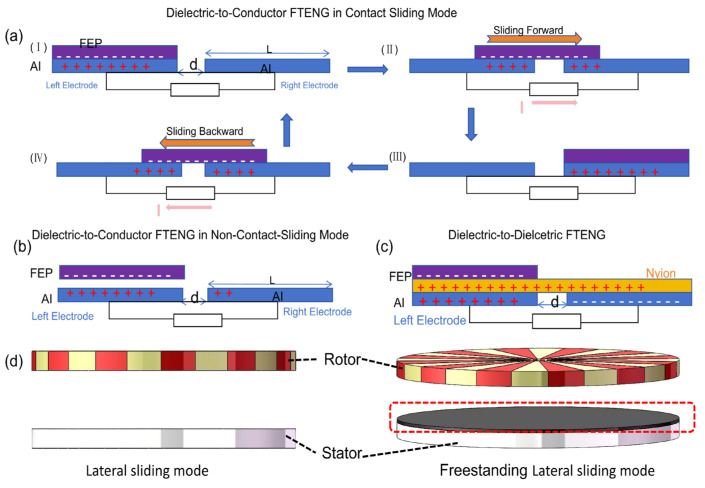
Working principle of Freestanding Triboelectric. Layer Mode (F-TENG): (**a**) Working principle diagram of conductor-dielectric F-TENG in sliding mode; (**b**) the working schematic diagram of the conductor-dielectric F-TENG in the non-contact sliding mode: (**c**) the schematic diagram of the conductor-conductor F-TENG; (**d**) Methods to distinguish F-TENG.

As the freestanding-layer moves between these electrodes, an uneven charge distribution is generated, inducing electron flow between the electrodes to neutralize local potential imbalances. The device structure and fundamental working principles of the freestanding-layer nanogenerator are depicted in Figure 4, with Figure 5a illustrating a typical conductor–dielectric configuration. In 2014, Wang et al. [61] pioneered the design and fabrication of the first freestanding-layer TENG model, specifically the sliding freestanding layer. Both numerical simulations and experimental observations revealed that this innovative model is capable of generating an output voltage surpassing 10 kV, with charge production that is directly proportional to the amount of triboelectric charge accumulated during each cycle.

The freestanding-layer Triboelectric Nanogenerator (F-TENG) operating mode, as depicted in Figure 5b, is characterized by the frictional charging of FEP, which enables it to swing between two electrodes, generating electricity without direct contact. This mode is viable because the charge induced by friction can be stored on the surface of the insulator for extended periods, ranging from hours to days. As long as the separation between the FEP and the electrodes remains sufficiently small (typically on the order of centimeters or more), even in the absence of charge movement on the FEP surface, sliding motion can still induce a charge flow. This unique F-TENG working principle distinguishes it from conventional TENG designs, as it operates without the need for physical contact. Based on this novel concept, Hao Zhang et al. applied a mixed hair TENG incorporating a brush TENG and a hair ball TENG, to enhance algae growth and establish a self-powered, autobiographical blue carbon ecosystem [62].

Furthermore, an alternative F-TENG design is based on the friction between two distinct dielectrics. In this configuration, FEP film serves as a freestanding layer, and nylon acts as an intermediate insulating layer that fully covers the two fixed electrodes, as illustrated in Figure 5c. When the FEP film comes into contact with the nylon surface, a negative charge is transferred from the nylon to the FEP surface. However, since the positive charge on the nylon surface is immobile, it cannot contribute to driving charge flow. Consequently, the movement of the negatively charged FEP film is the sole source of driving power for charge transfer. This principle mirrors the operation of the dielectric–conductor F-TENG structure, highlighting the analogous electricity generation mechanism in dielectric–dielectric configurations. Drawing on this principle, Heli proposed an innovative ocean-based device that combines vortex-induced vibration (VIV) and multi-grating TENG (MG-TENG) to harvest energy from low-speed ocean currents. This device is driven by the vertical motion of a float, converting kinetic energy into electrical power. The contact-free freestanding layer structure, which employs vertical charge separation, enables vibration between the two electrodes, inducing potential variations that generate current.

To further delineate the distinctions between the F-TENG and other TENG variants, Figure 5d demonstrates that the freestanding layer introduces an intermediate friction layer, eliminating direct contact between the positive and negative electrode layers. For example, in the disk sliding mode, the rotor and stator components are typically in direct contact, as shown on the left of the figure. This direct contact leads to significant frictional losses. To mitigate this issue, a friction layer is introduced between the rotor and stator, thus preventing direct contact. This modification, which incorporates a freestanding layer, provides distinct advantages, including increased motion flexibility and improved output efficiency. However, this design also introduces increased complexity, requiring precise control over the motion trajectory. In contrast, the contact-separation mode features a simpler structure but offers limited motion diversity. The sliding mode, while accommodating continuous motion, suffers from significant wear, and the single-electrode mode, though easy to integrate, typically results in lower output power. Of these four sliding modes, three are practically utilized in various applications. Specifically, in scenarios where one contact surface of the TENG cannot be electrically connected, the configuration is defined as the single-electrode mode.

## 3. Research Directions of TENG Application Extension, Structural Optimization, and Material Optimization

As a macroscopic manifestation of the coupling between the triboelectric effect and electrostatic induction, TENGs effectively showcase their potential to convert various dispersed energy sources into electrical energy across a wide range of scenarios [63]. Moreover, the electrical energy harvested by TENGs can serve as an indirect indicator of an object’s motion, thereby enabling the development of self-powered sensing systems. Due to their broad application prospects, TENGs have become a focal point of scientific research. In the following section, we provide a comprehensive and in-depth analysis of the current research landscape of TENGs, focusing on three key dimensions: the expansion of application scenarios, innovations in structural design, and the optimization of material properties. This analysis is based on the latest research frontiers and emerging development trends in the field of TENGs.

### 3.1. Application Extension

The original motivation behind the development of TENGs was to demonstrate the concept that “everything can generate electricity,” enabling their integration into diverse environments for the purpose of harvesting scattered ambient energy. With continuous advancements in this field, TENGs have been successfully applied across an increasing number of domains. In most environments, abundant yet untapped forms of dispersed energy exist, and TENGs play a critical role in realizing their effective collection and conversion.

**Figure 6:**
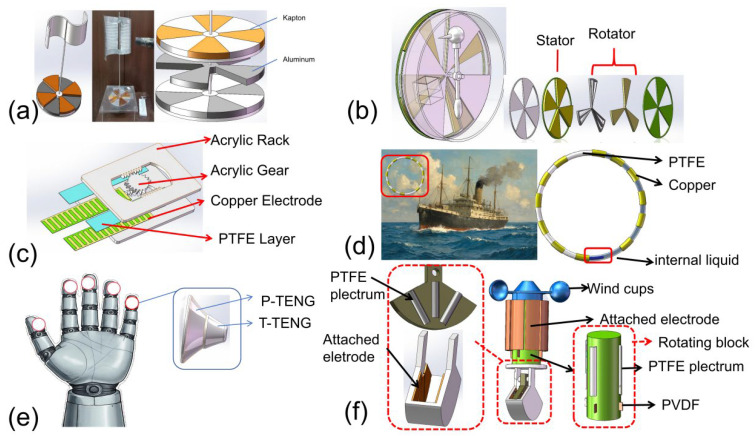
Application of TENGs in different places: (**a**) Schematic diagram of TENG used in the field of wind energy; (**b**) TENG used in the field of thermal energy; (**c**) Design drawings of TENG used in the mechanical field; (**d**) Structure diagram of TENG using wave energy; (**e**) Man–machine interaction; (**f**) Wave energy and wind energy mixing.

As illustrated in Figure 6a, Mehran Ali et al. [64] designed a wind-energy-harvesting TENG based on a Savonius vertical-axis wind turbine operating in a sliding mode. In this device, an acrylic sheet affixed to the turbine shaft serves as the upper triboelectric layer, while Kapton tape is employed as the static friction layer. Under a wind speed of 8 m/s, the device produced a short-circuit current (I_sc_) of 25 μA and an open-circuit voltage (V_oc_) of 40 V. The maximum output power reached 50 μW, corresponding to a power density of 110.58 W/m^2^, based on a contact area of 144 cm^2^.

Beyond wind energy, TENGs have also been explored for thermal energy harvesting. As shown in Figure 6b, Hang Qu et al. [65] proposed the first evaporation-induced TENG, which leverages environmental thermal energy. A drinking-bird-inspired heat engine was utilized to convert atmospheric water evaporation into mechanical motion, subsequently harvested by the TENG. The device achieved an open-circuit voltage of 382 V and a peak output power of 0.42 mW, which is three orders of magnitude higher than that of a droplet-based generator consuming the same amount of tap water, yielding an energy density of 59.7 mJ/mL.

In addition, TENGs have shown great potential in motion sensing applications. As depicted in Figure 6c, Senpeng Lin et al. [66] developed a self-powered rotational motion sensor (SRM-Sensor) capable of simultaneously monitoring displacement, speed, and acceleration. The device consists of rack-shaped acrylic plates and an incomplete gear, where rotary motion is transformed into linear reciprocating sliding. In sliding-mode operation, continuous electrical output is generated during rotation. The integration of a rectifier bridge and filter capacitor enabled a voltage amplitude correlated with rotational speed, while a symmetrical gear-rack structure amplified the triboelectric output signal twofold, achieving a sensitivity of 580 mV/rpm.

Furthermore, as shown in Figure 6d, Song Wang et al. [67] designed a robust self-powered inclination sensor based on an annular liquid–solid interface TENG for human–machine interaction. The sensor consists of a PTFE ring tube with copper electrodes on its surface, filled with a bubble-free liquid. By optimizing electrode width and liquid composition, the structural design significantly improved sensitivity. Durability tests confirmed stable output performance under low-frequency, low-amplitude tilt conditions. Importantly, due to the absence of mechanical moving parts, the sensor exhibited negligible wear and high durability, even in harsh environments characterized by high humidity, salinity, and strong magnetic fields. Experimental results revealed that both V_oc_ and Q_sc_ increased with the tilt angle, while distinct I_sc_ peaks occurred as the liquid column passed over the electrodes. The polarity of these peaks allowed for accurate determination of tilt direction. With its structural resilience and environmental robustness, this TENG-based inclination sensor provides a reliable solution for monitoring ship inclination, thereby further expanding the applications of self-powered sensing systems.

The multi-modal triboelectric sensor developed by Yanhua Liu et al. [68] demonstrates significant potential for advancing front-end human–computer interaction in skin-like sensors capable of detecting multiple stimuli simultaneously. However, achieving multimodal tactile recognition beyond the capabilities of human tactile perception remains a considerable challenge. To address this, a multi-modal triboelectric sensor with adaptive functionality in extreme environments is proposed, enabling the detection of pressure and temperature beyond the limits of human perception. This sensor utilizes TENGs and features an asymmetric structure that outputs dual signals independently, thereby enhancing the sensitivity of the sensor. By converting the detected signals and stimuli into a characteristic matrix, the sensor achieves parallel sensing of complex objects (with a recognition rate of 94%) and high-temperature detection. This system represents progress in terms of both the detection range and response speed, achieving the upper limit of human skin’s thermal sensing ability (60 C) with an operational temperature range extending to 200 C. The proposed self-powered multi-modal sensing system opens up new possibilities for applications in human/robot/environmental interactions.

In a separate development, Jianping Li et al. [69] introduced a hybrid energy harvesting device based on piezoelectric TENGs, capable of simultaneously capturing wind and wave energy. This device incorporates three distinct energy collection modules: a wind energy collection triboelectric nanogenerator (WD-TENG), a wind energy collection piezoelectric nanogenerator (WD-PENG), and a wave energy collection triboelectric nanogenerator (WE-TENG). These modules can function independently or in tandem, enhancing the technical reliability of micro-nano energy harvesting systems in unstable environments such as the ocean. In simulations, each module produced 3.975 mW, 1.160 mW, and 0.2925 mW of power, with corresponding power densities of 5.064 W/m^3^, 1.478 W/m^3^, and 1.092 W/m^3^, respectively. This work underscores the promising applications of TENG technology in energy collection systems, particularly in challenging environments.

As these examples illustrate, TENGs are increasingly being applied across diverse fields. The growing body of research on TENG signals an expansion of their application range. As the field continues to evolve, researchers are not only focusing on singular applications but also exploring cross-disciplinary approaches. This broadens the scope of current TENG research and points toward exciting future directions for the technology, as shown in Table 3. As the technology matures, it is anticipated that TENGs will penetrate even more sectors, potentially unveiling new applications previously unimagined.

**Table 3:** Application of TENG in Different Fields.

Field	Structure Lype	Output Performance	Ref.
Blue Energy	Rolling Spherical Structure	7.96 mW, 120 μA, 560 V, 15.20 Wm^−3^	[69]
wind	Contact-separation	300 V, 12 μA, 200 nC	[70]
	Contact-separation	400 V, 7 μA, 80 nC	[71]
Human skin	Single electrode	10 Pa, 1860 V, 1.1 μA/cm^2^ 5200 mW/m^2^, 5.09 mW/N	[72]
	Single electrode	9.8 Pa, 28 V, 0.56 μA	[73]
Machine learning	four-layer GNN	1.12 J/cm	[74]
ground motion	Contact-separation	Minimum traction and compression forces of 35 N at minimum velocities of 10 mm/min for elongations up to 4 mm could be detected	[75]
Healthcare	Single electrode	14.5 W m^2^ 85 μA	[76]
Pervskites	Contact-separation	17 V, 30 μA, 130 μW, 14.44 μW/cm^2^	[77]
Mechanical energy	cylindrical rollers	26.56 V, 2.45 μA	[78]
optics	Single electrode	achieving remarkable elasticity over 100% and a brightness of 139 cd/m^2^.	[38]
Unmanned aerial vehicle	Contact-separation	with a wide frequency detection range of 20–400 Hz, a maximum error of 0.0062%, and a linear fit goodness of fit (R^2^) close to 1.F	[79]

### 3.2. Structure Optimization

**Figure 7:**
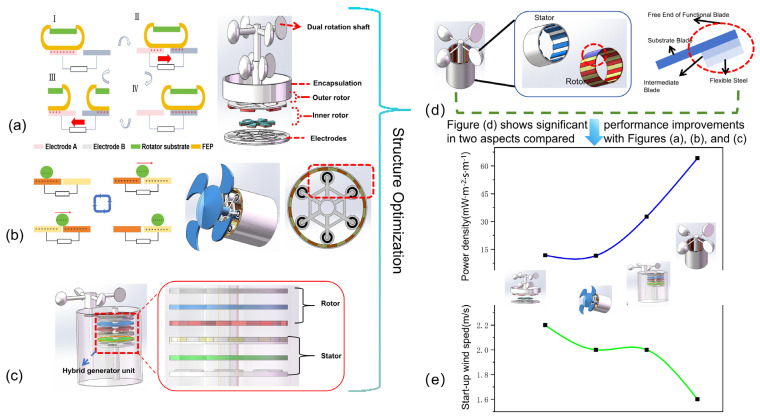
Application of wind energy: (**a**) Charge excitation and mode adjustable triboelectric nanogenerator structure principle (CEMA-TENG); (**b**) Planetary rolling triboelectric nanogenerator structure and working principle (PR-TENG); (**c**) Travel-controlled triboelectric nanogenerator structure and working principle (TC-TENG); (**d**) Structural diagram of wind speed adaptive triboelectric nano generator optimized on the basis of figure (**a**–**c**) (WSA-TENG); (**e**) Comparison of “Startup Wind Speed” and “Peak Power Density” for figures (**a**–**d**).

As research on TENG continues to advance, an increasing number of application scenarios are being developed. In established fields, the focus of research can shift from expanding the scope to deepening the understanding of TENG, particularly through efforts aimed at improving power generation efficiency and other performance metrics. One effective approach to achieving these improvements is through structural optimization. For instance, in the context of wind energy applications, there is significant potential to explore the optimization of TENG structures. As illustrated in Figure 7, we present a TENG structure specifically designed for wind energy applications, demonstrating how performance enhancements in TENG can be realized through strategic structural optimization.

During the course of our research, we discovered that TENGs not only enable the realization of the concept that “everything can be triboelectrically charged” through their diverse, cross-disciplinary applications but also offer substantial potential for iterative structural design and optimization. In particular, by focusing on wind energy applications, we explored various design improvements aimed at enhancing TENG performance and broadening their scope of use.

The invention of the CEMA-TENG by Shaoke Fud et al. [80], as illustrated in Figure 7a, demonstrates its capability to harvest energy from water flow and monitor smart forests. The structure of the CEMA-TENG consists of two primary components: the upper section, which functions as the generator, and the lower section, which serves as a water wheel. The water wheel collects energy from flowing water, particularly from mountainous regions, driving the rotation of the CEMA-TENG’s rotor. The stator and rotor in this TENG can automatically make contact at low speeds and separate at high rotational speeds, ensuring that the rotor consistently operates at lower speeds. This gear system effectively reduces material surface wear. The CEMA-TENG demonstrates remarkable stability, maintaining 94% of its electrical output after 72,000 cycles, significantly outperforming conventional contact-based TENGs, which retain only 30% of their output. Thanks to its high electrical stability and substantial electrical output, the CEMA-TENG is capable of powering 944 green light-emitting diodes (LEDs) connected in series. Additionally, by harvesting water flow energy, the system enables rapid charging of various commercial capacitors and supports self-powered fire alarm systems and temperature-humidity monitoring devices. This work presents an ideal solution for enhancing the mechanical durability of TENGs, extending their operational frequency range, and improving electrical output.

Figure 7b [81] presents the design of a breeze-driven, autonomous wireless wind anemometer (WWA) based on a planetary rolling triboelectric nanogenerator (PR-TENG) for simultaneous wind energy harvesting. The PR-TENG operates in a rolling self-supporting mode and is equipped with six pairs of finger-like copper electrodes. As shown in the figure, the planetary frame rotates counterclockwise when driven by the breeze, while the roller rotates clockwise around its axis. The planetary rolling structure facilitates rolling motion between the two triboelectric layers, effectively reducing friction. The working principle of the PR-TENG, which combines contact electrification and charge transfer induced by in-plane rolling, is depicted on the right side of Figure 7b. Due to the characteristics of planetary rolling friction, the PR-TENG can be activated at wind speeds as low as 2 m/s. At wind speeds of 5 m/s, the WWA system can continuously supply power and autonomously transmit wind speed data within a 10-m range every 2 min.

As shown in Figure 7c [82], this paper proposes an innovative stroke control method that effectively reduces mechanical wear by combining a gear mechanism with a cam switch. This method enables automatic switching between contact and non-contact modes, with the switching frequency adjustable. After continuous operation for 80 h (equivalent to 1.92 million cycles), the system maintains 90% of its electrical energy output, demonstrating excellent electrical stability. Furthermore, through structural optimization, the power density per unit wind speed of the stroke-controlled triboelectric nanogenerator (TC-TENG) has been improved by 100% compared to previous related research. Additionally, this study integrates a triboelectric-electromagnetic hybrid device with an energy management circuit, successfully developing a self-powered closed-loop environmental monitoring and alarm system. In a breeze environment (wind speed less than 3 m/s), this system can continuously and stably monitor environmental conditions and transmit data to mobile devices through wireless transmission technology. The structure and working principle of the hybrid device are shown in the overall structural schematic diagram. The core of the device is a wind-driven hybrid nanogenerator, combining TC-TENG with an electromagnetic generator (EMG). Key components include a wind cup, a housing, a transmission device, a cam switch, and a hybrid generator set. The transmission device consists of a transmission shaft and a gear train, which transmits part of the wind energy captured by the wind cup to the cam switch. During rotation, the cam switch achieves flexible switching between contact and non-contact modes by adjusting the height of the stator shaft.

It has been observed that achieving both high power density and low starting wind speed simultaneously is challenging for the three TENG types shown in Figure 7a–c. To overcome this limitation, a novel WSA-TENG has been developed, as shown in Figure 7d [83], offering improved performance compared to previous designs. This new system operates at lower starting wind speeds, while simultaneously achieving higher power density and significantly reducing material wear. The performance comparison is shown in Figure 7e. The WSA-TENG consists of a stator, rotor, and functional blades. The rotor includes a foam cylinder, an intermediate sponge, a substrate sponge, 34 copper electrodes, and a pair of flexible magnets. The intermediate sponge is affixed to the outer surface of the foam cylinder, with the copper electrodes uniformly distributed on its surface. The substrate sponge is placed on the outer surface of the foam cylinder, with its curvature adjusted to ensure a flat fit for the flexible magnets on the substrate’s outer surface. The stator consists of an acrylic cylinder with 40 triboelectric thin films (20 FEP and 20 nylon films) arranged alternately along the inner surface. The functional blades are composed of a base plate blade, an intermediate blade, and flexible steel, with the intermediate blade and flexible steel fixed to the free end of the base plate.

The WSA-TENG, through optimized structural design, operates in a non-contact mode at low wind speeds and in a soft-contact mode at higher wind speeds. This design significantly reduces the starting wind speed (only 1.6 m/s), enhances durability, and increases power density, making it ideal for practical wind energy harvesting. At a wind speed of 3.3 m/s, the WSA-TENG achieves a peak power density of 64.2 mW/m^3^. Furthermore, after 90,000 cycles of continuous operation, its power output retains 99.4% of the initial value, demonstrating exceptional durability.

This study underscores significant progress in TENG technology, particularly with respect to improvements in mechanical durability, power output, and energy harvesting efficiency. These developments not only promise to elevate the potential of TENGs as a viable energy source but also pave the way for more efficient and sustainable renewable energy systems in the future. In addition to the advancements in the mechanical structure of the TENG, optimizing the material properties also plays a pivotal role in enhancing its performance.

### 3.3. Material Optimization

**Figure 8:**
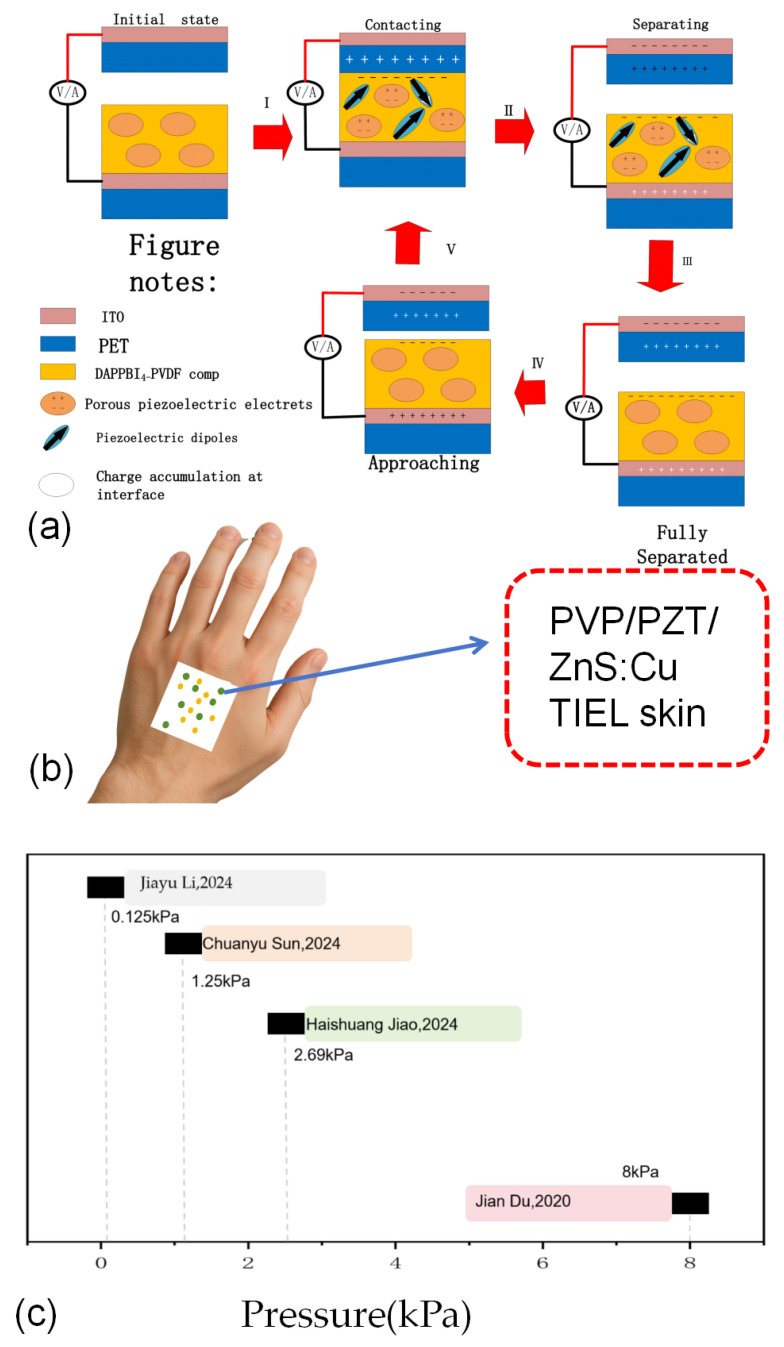
(**a**) chemically adding a friction layer and a generator electrode in a vertical contact-separation mode; (**b**) Schematic diagram of a single-electrode TNEG acting on human body in the research field; (**c**) Comparison of pressure in papers [45,84,85,86] with different chemical additives in the same field and structure.

In addition to structural optimization, there is also a chemical treatment of generator materials when optimizing generators. As shown in Figure 8.

When studying the materials of TENGs, the vertical contact out formula with simple structure is usually selected as shown in Figure 8a. Perovskite materials are considered as a promising candidate for TENG because of their excellent dielectric properties [83]. Take perovskite as an example. In order to increase the efficiency of perovskite as a power generation layer, the experimenter will process it twice. Swathi Ippili et al. [87] selected two-dimensional layered halide perovskites with excellent piezoelectric/ferroelectric properties and superior environmental stability as experimental subjects. They introduced 3-propane diamine lead iodide (DAPPbI_4_) perovskite as a filler into polyvinylidene fluoride (PVDF) to develop a DAPPbI_4_-PVDF composite TENG with high performance and stable mechanical strength. By comparing the different contents of DAPPbI_4_, it is concluded that the triboelectric output performance of 15 wt% DAPPbI_4_ is significantly enhanced, which is attributed to its improved electroactive β-phase PVDF (~90%), high dielectric constant (~47.4) and high piezoelectric coefficient (~26.6 pm/V). The TENG achieves a high output voltage of ~662 V, a current density of ~18.7 μA/cm^2^, an unprecedented high power density of ~4.28 m W/cm^2^, and an excellent pressure sensitivity of ~13.31 V/kPa, and has excellent mechanical durability and operational stability. For example, Xiangcong He et al. added Cs_2_Ag_0.3_Na_0.7_InCl_6_ into perovskite and formed a high-performance structure with PVDF. PENG based on Zr/Te codoped CDP @ PVDF has excellent piezoelectric output, and its maximum open-circuit voltage and short-circuit current density are 67 V and 18 μA/cm^2^, respectively. This performance level is ~19 and ~12 times higher than PVDF-based PENGs without CDP.M. Sahu et al. [88] designed a triboelectric nanogenerator (TP-TENG) that operates in a vertical contact-separation mode. It exhibits an electrical response of 300 V and 2.2 mA to a10 wt% polydimethylsiloxane-Sr_3_Co_2_WO_9_ (PDMS-SCWO) composite film. The power density of the rough-surface TP-TENG is 30.5 mW/cm^2^, significantly higher than that of the smooth-surface TP-TENG, which has a power density of 5.5 mW/cm^2^. Other details are shown in Table 4.

**Table 4:** Several kinds of TENG with different compositions based on perovskite.

Perovskite	*V*_OC_	I_sc_	Refs.
$Ba(Cu_{0.5}W_{0.5})O_{3}(BCW)+PMDS$	127 V	3.16 μA	[89]
$CsPbBr_{2.6}I_{0.4}$	192 V	16.7 μA	[90]
V-NaNb$o_{3}$	−200 V	−5.7 μA	[91]
FTO/CsPbB$r_{3}$	240 V	4.13 μA	[92]
$DAPPBI_{4}-PVDF$	~662 V	~18.7 μA	[87]
$Cs_{0.05}FA_{0.7}MA_{0.25}$ Pb$I_{3}$	200 V	16.3 μA	[93]
$Cs_{2}Ag_{0.3}Na_{0.7}InCl_{6}$	67 V	18 μA	[94]
CsFAMA	0.33 V	2.1 μA	[95]

After conducting numerous experiments on TENGs, researchers have found that, in many cases, the materials used are highly similar or even identical, with polyvinylidene fluoride (PVDF) being a common choice. PVDF exhibits good resistance to most chemicals and maintains stable performance in acidic, alkaline, or humid conditions. This characteristic significantly enhances the reliability and service life of TENG devices in different environments, thereby broadening their application scope. It can be said that the excellent performance of PVDF has provided an important driving force for the rapid development of TENG research. However, as the number of TENG application cases continues to increase, researchers have observed significant differences in the power generation performance of TENGs when using PVDF with different compositions. This discovery has prompted a shift in research direction, with researchers focusing on the PVDF material itself and exploring its optimization potential in TENGs.

For example, in paper [95], electrospun polyvinylidene fluoride-hexafluoropropylene (PVDF-HFP) composite nanofiber films were utilized as the triboelectric negative layer. By incorporating a combination of conductive silver nanowires (AgNWs) and perovskite oxide Mn-doped (B_i0.5_Na_0.5_)TiO_3_-BaTiO_3_ (Mn-BNT-BT) nanocrystals into electrospun PVDF-HFP nanofibers, a significant increase of 386% in the output power of the original PVDF-HFP nanogenerator was achieved. For the hybrid TENG containing 5% AgNWs and 5% Mn-BNT-BT nanocrystals, a peak open-circuit voltage of 2170 V and a power density of 47 W/m^2^ were achieved, which significantly surpassed the output performance of previous PVDF-based TENGs. A comparison with other PVDF-based TENGs is shown in Table 5.

**Table 5:** Comparison of output performance of this work against PVDF-based TENG in the literature.

Materials	Open-Circuit Voltage (V)	Peak Power Density (W/cm^2^)	Ref.
PVDF-HFP+Mn-BNT-BT + AgNWs composite fiber mat/Al foil	2172	47.3	[96]
PVDF/ZnO NWs	330	3	[97]
PVDF/MXene nanocomposite fiber	710	11.213	[98]
PVDF nanofiber mat/conductive fabric	400	7	[99]
PVDF/printer ink (PI) nanocomposite fiber	1600	22	[85]
PVDF-MoS_2_/CNTs nanocomposite fiber	300	0.134	[86]
PVDF film /FTO/Co(OH)(CO_3_)0.5/Pt/CsPbIBr_2_	243	2.04	[100]

In the research of TENGs, polyvinylidene fluoride (PVDF) is often used as the negative electron layer (i.e., the electron acceptor layer in the triboelectric layer). Meanwhile, the other triboelectric layer is usually paired with different materials to observe the changes in current and voltage when these materials come into contact with PVDF. This method enables a more accurate screening of ideal positive electron layer (electron donor layer) materials. Based on this, by fixing the positive and negative electron layers, the performance of materials in TENGs can be further precisely evaluated and optimized. Here, we take PVDF and SrTiO_3_ as examples. In paper [86], flexible piezoelectric films of polyvinylidene fluoride (PVDF) and novel perovskite SrTiO_3_ (ST) were prepared using a highly scalable supersonic cold spray technology. Due to the hydrothermal synthesis of SrTiO_3_ nanocubes and the supersonic process, a huge shear stress was applied to PVDF during the cold spraying process, resulting in a film with an effective piezoelectric coefficient of 69.6 pm/V. The piezoelectric nanogenerator, with a load resistance of 0.9 MΩ, generated a maximum power of 130 μW, as confirmed by a piezoelectric force microscope. Under an external force of 20 N and a frequency of 7 Hz, the composite film exhibited durability for 21,000 tapping cycles. The flexural endurance was determined from 3000 bending cycles. The TENG attached to the knee joint provided voltages of 1 V and 2.3 V when bent to 45° and 90°, respectively. After polarization, the TENG generated a piezoelectric potential of 31 V under a tapping force of 20 N. Compared with other technologies, such as those listed in Table 6, the flexible TENG and piezoelectric composite film deposition technology (cold spraying) were superior to several other titanate-based TENGs.

**Table 6:** Comparison of TENGs of SrTiO3/PVDF.

Material	V_oc_ (v)	I_sc_ (μA)	Power (μW)	Power Density [W/cm^2^]	References
PZT/MFC@PVA	16.5	0.86	3.3	1.5	[86]
ZnTiO_3_/PDMS	6.5	0.07	1.43	2.86	[101]
Bi_0.5_Na_0.5_TiO_3_/PVDF	19	1.2	1.4	0.35	[86]
CaTiO_3_/PVDF	12	0.1	1.71	0.19	[102]
BaTiO_3_/PVDF	25.7	0.68	-	-	[103]
BSTO-MWCNTs/PVDF	42	9	31.5	31.5	[104]
BaTiO_3_/PVDF	24.5	0.64	0.7	0.4	[105]
SrTi2O_5_/PDMS	~10	0.92	0.64	0.16	[106]
SrTiO_3_/PVDF	17	30	130	14.44	[78]

In addition to similar situations in the perovskite field, new applications have also emerged in other areas, such as designs for human body sensing. Jiayu Li et al. [84] (as shown in Figure 8b) proposed an innovative solution to address the shortcomings of complex structures, external power sources, or strong mechanical stimuli required during the excitation process of light-emitting devices. Based on existing experiments, they cleverly introduced PZT (lead zirconate titanate) powder as a component, spin-coated an appropriate amount of the mixture onto a polyvinyl chloride (PVC) substrate, and prepared a novel triboelectric-induced electroluminescence (TIEL) skin. Experimental results showed that the introduction of the matrix PVP (polyvinylpyrrolidone) and Pb(Zr_x_Ti_1x3_Ti_1x3_O_x_) (PZT) significantly enhanced the dielectric properties and polarization ability of the skin, thereby greatly improving its triboelectric performance and luminous intensity. The pressure threshold of the TIEL skin was reduced to a record-breaking 0.125 kPa, showing significant advantages compared to other similar research results (as shown in Figure 8c).

Researchers have leveraged the inherent properties of materials and applied them to TENG. By adjusting the composition ratios or introducing new materials to prepare composite materials, they have significantly enhanced the performance of TENGs. Taking PVDF as an example, its widespread application has not only driven the development of TENG technology but also enabled researchers to deeply investigate the material properties of PVDF through the characteristics of TENG. Furthermore, research on composite materials such as SrTiO_3_/PVDF, by precisely controlling the proportions of the two materials, has revealed the impact of material composition on the power generation efficiency of TENG, highlighting the crucial role of material optimization in enhancing device performance. To some extent, TENG are not only energy harvesting devices but also serve as an effective tool for evaluating the discharge capacity of materials. Looking ahead, TENGs are poised to become an important testing method in materials science, supporting the development of novel functional materials. Researchers have innovatively applied the inherent properties of materials to TENG by adjusting the proportions of different components or introducing new elements to prepare composite materials, effectively enhancing the performance of TENG. Taking PVDF (polyvinylidene fluoride) as an example, its excellent piezoelectricity and flexibility have led to its widespread use, not only driving the development of TENG technology but also enabling researchers to deeply explore the material properties of PVDF through the characteristics of TENG. Further research on composite materials such as SrTiO_3_/PVDF, by precisely controlling the proportions of the two materials, has revealed the impact of material ratios on the power generation efficiency of TENGs, highlighting the crucial role of material optimization in performance enhancement. In a sense, TENGs are not only energy harvesting devices but also serve as an effective tool for evaluating the discharge capacity of materials. Looking ahead, TENGs are poised to become an important testing method in the field of materials science, supporting the development of novel functional materials.

## 4. Conclusions and Extensions

**Figure 9:**
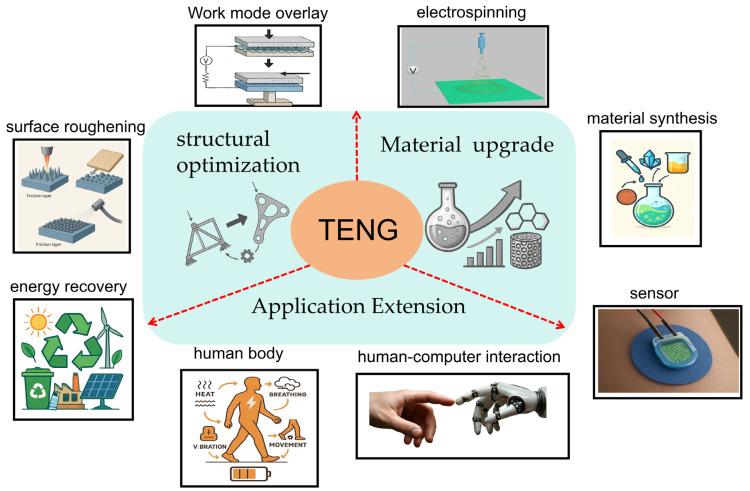
TENG structure optimization and material updates drive the development of future fields.

Since its inception in 2012, TENGs have emerged as a key energy harvesting technology, capable of efficiently converting mechanical energy into electrical energy. TENGs have found widespread applications in diverse fields, including wearable devices, the Internet of Things (IoT), and environmental monitoring. As illustrated in Figure 9, the development of TENG technology has catalyzed innovative trends in modern energy harvesting and applications, particularly through advancements in structural optimization and material selection. Notably, the adaptability and efficiency of TENGs in complex environments have driven their success. By integrating various energy harvesting mechanisms such as wind, wave, and frictional energy, TENGs can operate either simultaneously or independently with multiple energy sources, thereby significantly enhancing energy harvesting efficiency, while improving the overall system stability and reliability.

Despite the significant progress achieved in laboratory settings, TENGs continue to face several challenges in their practical deployment. These challenges include enhancing energy harvesting efficiency, optimizing structural designs, selecting suitable materials, and addressing compatibility issues across different energy sources. Future research should prioritize the integration and optimization of multiple energy sources, steering the development of TENGs toward more intelligent and adaptive systems. Concurrently, efforts should be made to advance the research and development of high-performance triboelectric materials, with an emphasis on improving their stability, wear resistance, and expanding their use in flexible and wearable devices. Furthermore, the multifunctional integration of TENGs and their systematic design will not only enable efficient energy harvesting but also facilitate real-time monitoring and data feedback. This would allow for broad applications in fields such as smart homes and health monitoring.

To address the challenges related to energy harvesting efficiency and stability, future research should also focus on the development of efficient energy management and storage technologies. The integration of supercapacitors and lithium batteries, for example, can help regulate energy fluctuations, thereby enhancing the continuous and stable operation of TENG-based systems. With ongoing advancements in materials science, structural design, and energy management technologies, TENGs are poised to have significant applications across various sectors, including environmental monitoring and healthcare. As such, TENGs are expected to play a crucial role in the promotion of sustainable development and the advancement of intelligent technologies in the near future.

## Data Availability

Not applicable.
